# Polymer Composite Design Strategies for Antibacterial Resin-Based Dental Restorative Composites: Mechanisms, Structure–Property Relationships, and Translational Challenges

**DOI:** 10.3390/polym18172147

**Published:** 2026-09-02

**Authors:** Chuan-Chi Chen, Tsu-I Yang, Yi-Chia Chen, Kuan-Wei Lung, I-Ta Lee, Tzu-Yu Peng, Jie-Ru You, Thi Thuy Tien Vo, Yung-Li Wang, Chien-Fu Tseng

**Affiliations:** 1 School of Dentistry, College of Oral Medicine, Taipei Medical University, Taipei 110301, Taiwan; b202114021@tmu.edu.tw (C.-C.C.); b202114052@tmu.edu.tw (T.-I.Y.); b202114006@tmu.edu.tw (Y.-C.C.); b202114057@tmu.edu.tw (K.-W.L.); itlee0128@tmu.edu.tw (I.-T.L.); typeng@tmu.edu.tw (T.-Y.P.); b202111019@tmu.edu.tw (J.-R.Y.); cetuswang@tmu.edu.tw (Y.-L.W.); 2 Faculty of Dentistry, Nguyen Tat Thanh University, Ho Chi Minh City 700000, Vietnam; vtttien@ntt.edu.vn; 3 Tseng Department of Dentistry, Taoyuan General Hospital, Ministry of Health and Welfare, No. 1492, Zhongshan Road, Taoyuan District, Taoyuan 33004, Taiwan; 4 Research Center for Tooth Bank and Dental Stem Cell Technology, College of Oral Medicine, Taipei Medical University, Taipei 110301, Taiwan; 5 Graduate Institute of Clinical Dentistry, School of Dentistry, College of Medicine, National Taiwan University, Taipei 100229, Taiwan

**Keywords:** antibacterial dental composites, polymer network design, quaternary ammonium methacrylates, bioactive fillers, secondary caries

## Abstract

Dental resin composites are widely used for direct restorations, yet their longevity remains limited by biofilm accumulation and secondary caries at the tooth–restoration interface. Unlike previous reviews that have primarily organized antibacterial dental materials according to antimicrobial agents or mechanisms, this narrative review evaluates antibacterial resin composites from a polymer-composite design perspective, integrating molecular architecture, network immobilization, filler–matrix interactions, polymerization, and aging. Leachable agents such as chlorhexidine provide early antibacterial effects but are constrained by reservoir depletion, water sorption, and release-related material changes. In contrast, covalently immobilized quaternary ammonium monomers provide sustained surface-associated activity without continuous release, although their performance depends on molecular structure, concentration, degree of conversion, and network properties. Antibacterial nanoparticles and bioactive glass fillers provide composition-dependent ion-mediated, photocatalytic, pH-modulating, and remineralizing effects, while their performance depends strongly on particle characteristics, dispersion, and formulation. Multifunctional systems further combine antibacterial activity with protein repellence, mineral protection, and rechargeable ion release. Overall, the evidence indicates that durable antibacterial performance cannot be considered independently of polymerization, mechanical integrity, aging stability, and biocompatibility. Future development should therefore prioritize clinically relevant multispecies biofilm models, standardized aging protocols, structure–property analysis, and long-term in vivo and clinical validation.

## 1. Introduction

Resin-based composites are widely used in restorative dentistry because of their favorable esthetics, adhesive properties, and compatibility with minimally invasive treatment. These materials generally consist of an organic resin matrix, inorganic fillers, silane coupling agents, and a polymerization initiator system. Most direct resin composites are light-cured, whereby visible-light activation of the photoinitiator system initiates polymerization of the resin matrix and formation of the crosslinked polymer network [1]. Advances in resin formulation and filler technology have improved their mechanical, optical, and handling properties [1]. Nevertheless, the longevity of composite restorations remains a clinical concern, and caries adjacent to an existing restoration is an important reason for repair or replacement [2,3].

Secondary caries is a multifactorial process influenced by patient-related caries risk, dietary exposure, oral hygiene, biofilm activity, restoration quality, and marginal integrity [4,5]. Resin composites do not directly cause secondary caries, but their material properties may influence the conditions at the tooth–restoration interface. Polymerization shrinkage can generate interfacial stress and marginal defects, whereas incomplete conversion may leave residual monomers within the polymer network. During clinical service, water sorption, hydrolytic degradation, filler–matrix debonding, surface wear, and increasing roughness may progressively alter the restoration surface and its margins [1,3]. Conventional resin composites also have little intrinsic antibacterial activity and provide limited protection against biofilm-related acid production [3,6].

Following exposure to the oral environment, salivary components adsorb onto the composite surface and facilitate bacterial attachment and biofilm development. Acidogenic biofilms can subsequently promote demineralization of enamel and dentin near restoration margins [6,7]. These limitations have encouraged the development of resin composites that interfere with bacterial adhesion, viability, metabolism, or acid production. Antibacterial modification is intended to reduce biofilm activity while maintaining the mechanical and esthetic properties required for long-term restorative use.

Antibacterial resin composites can be broadly classified as leachable, contact-active, or multifunctional systems [6,8]. Leachable systems contain soluble antibacterial agents that diffuse from the resin matrix or carrier particles. Chlorhexidine (CHX)-containing composites can provide substantial initial antibacterial activity, but their effectiveness may decrease as the available reservoir is depleted. Agent release may also be associated with water uptake, pore formation, and deterioration of material properties [9,10,11,12]. Contact-active systems instead use polymerizable antibacterial monomers or immobilized polycations that become incorporated into the cured resin network. Quaternary ammonium methacrylates (QAMs), including 12-methacryloyloxydodecylpyridinium bromide (MDPB) and dimethylaminoalkyl methacrylates, provide surface-associated antibacterial activity without relying on continuous release [8,13,14,15,16]. Their performance is influenced by molecular structure, charge density, concentration, polymerization behavior, and compatibility with the resin matrix.

Multifunctional systems combine antibacterial activity with additional properties such as protein repellence, calcium and phosphate release, bioactivity, or ion rechargeability [17,18,19,20]. These materials are designed to interfere with bacterial attachment and biofilm growth while also reducing acid-related mineral loss. However, combining multiple functional components requires careful balancing of antibacterial and bioactive functions with overall material performance [6]. The biological benefit of an antibacterial component must therefore be considered together with its effects on polymer-network formation and long-term material performance.

Most evidence for antibacterial resin composites remains derived from in vitro studies using standardized specimens and simplified biofilm models [4,6,8]. These models are useful for evaluating antibacterial mechanisms but do not fully reproduce the effects of saliva, dietary pH changes, mechanical loading, surface wear, and restoration-margin geometry. Clinical evidence supporting the long-term prevention of secondary caries by antibacterial resin composites remains limited [6,8]. Antibacterial activity under laboratory conditions should therefore not be regarded as sufficient evidence of clinical effectiveness.

Previous reviews have commonly organized antibacterial dental materials according to antimicrobial agents or mechanisms and summarized their biological and material properties [6,8]. The present review examines antibacterial dental composites from a polymer-composite design perspective, with emphasis on release behavior, covalent immobilization, molecular structure, filler–matrix interactions, polymerization, aging, and multifunctional integration. Leachable, contact-active, and multifunctional systems are evaluated in relation to their antibacterial mechanisms, structure–property relationships, biological safety, durability, and potential for clinical translation.

## 2. Literature Search Strategy

A narrative literature search was conducted to identify publications on antibacterial dental resin composites, with emphasis on polymer design, antimicrobial mechanisms, material properties, aging, and clinical translation. PubMed, Scopus, and Web of Science were searched from database inception to 28 July 2026. The search combined terms related to dental resin composites with terms describing antibacterial agents and polymer systems, including “dental resin composite,” “antibacterial composite,” “antimicrobial resin,” “quaternary ammonium methacrylate,” “MDPB,” “DMAHDM,” “chlorhexidine,” “silver nanoparticle,” “zinc oxide,” “titanium dioxide,” “bioactive glass,” “calcium phosphate,” “protein-repellent polymer,” “remineralization,” “degree of conversion,” “water sorption,” and “aging.” The reference lists of relevant reviews and selected articles were also screened to identify additional publications.

**Figure 1:**
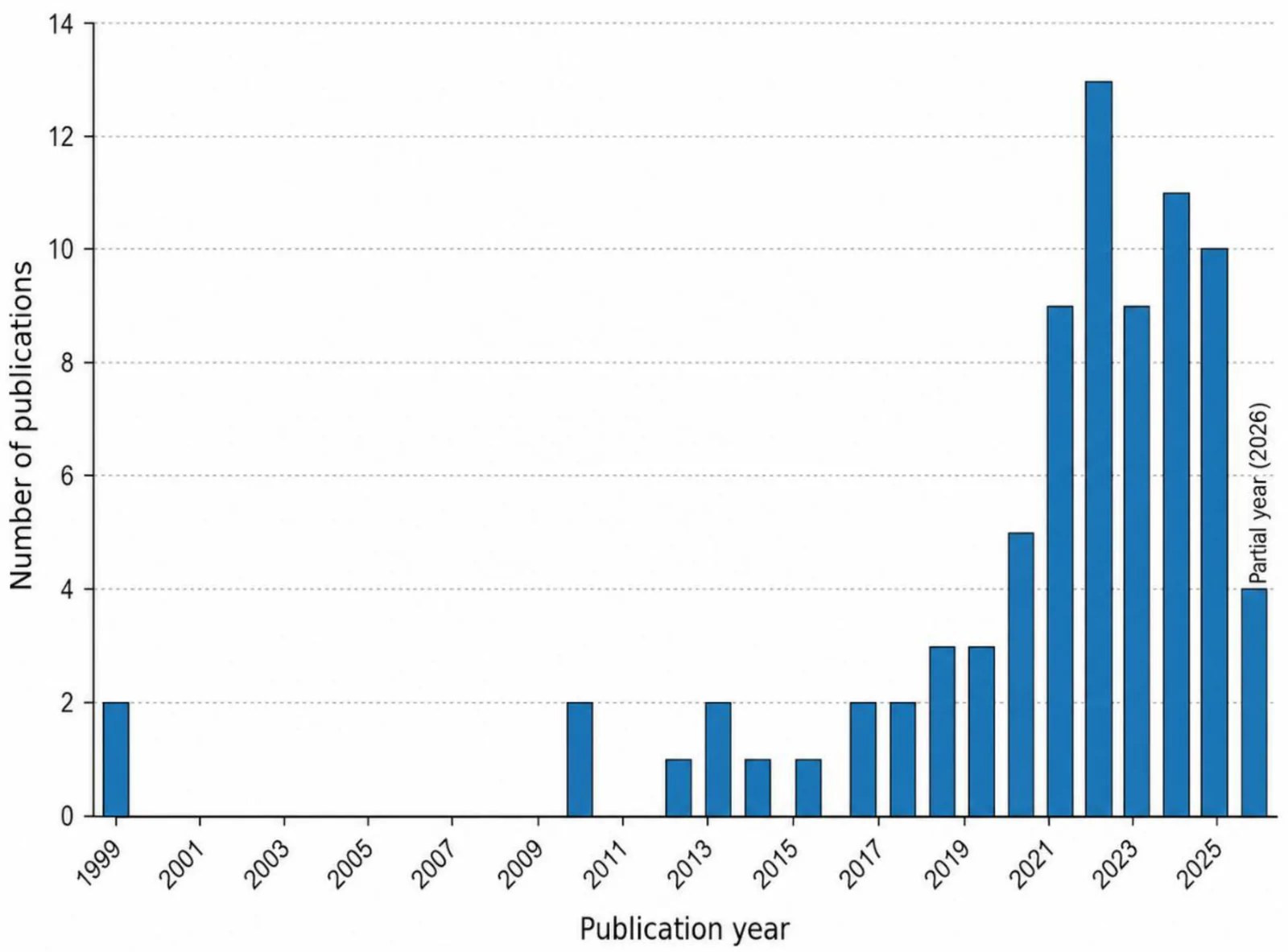
Annual publication activity on antibacterial resin-based dental restorative composites identified in PubMed. The figure shows the number of publications per year retrieved using the predefined PubMed search strategy and retained after manual screening for relevance to antibacterial resin-based dental restorative composites. A total of 80 publications were included. Data for 2026 represent a partial year up to 28 July 2026.

**Figure 2:**
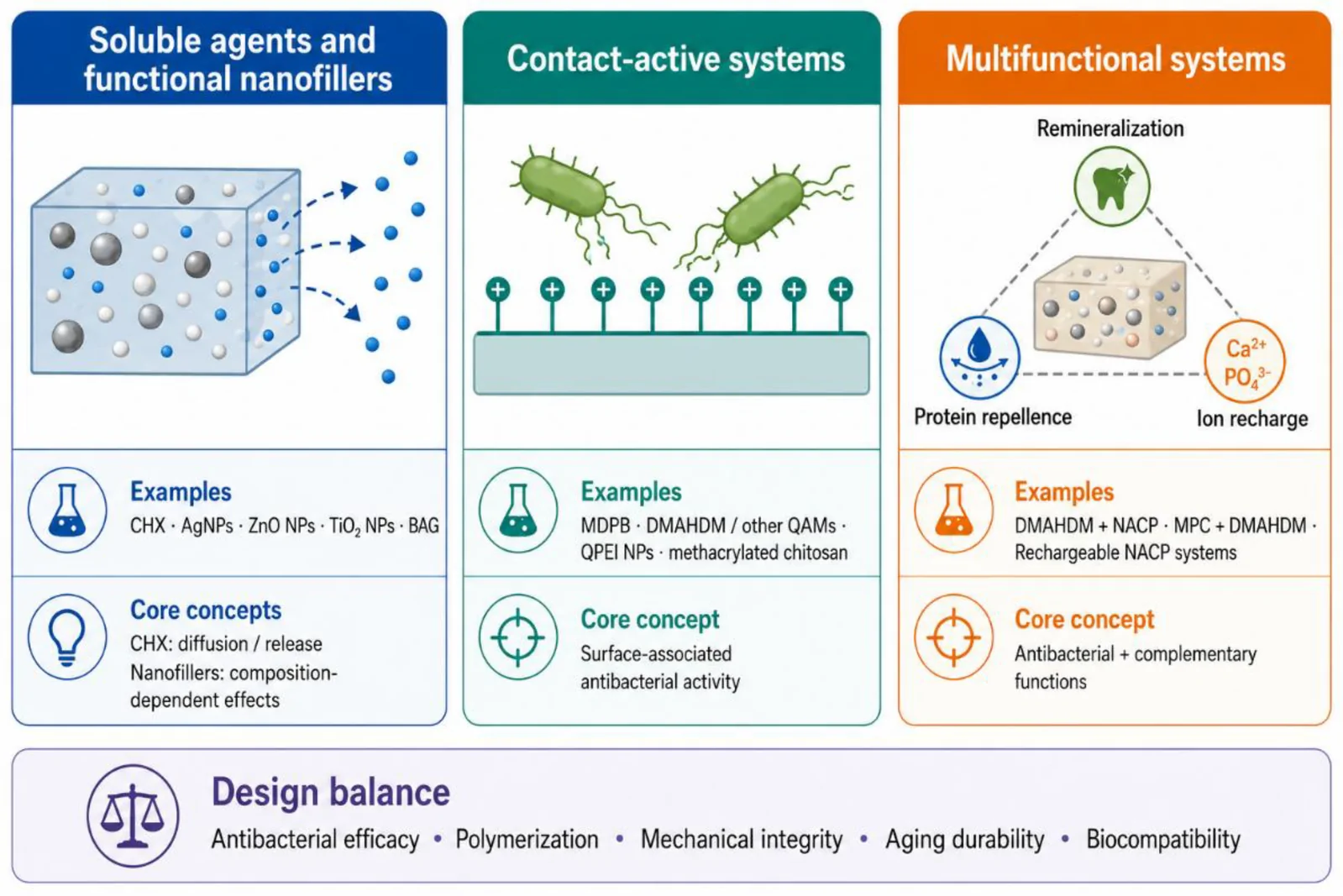
Polymer-composite design framework for antibacterial dental resin composites. Soluble agents and functional nanofillers include chlorhexidine (CHX), which acts primarily through diffusion and release, and functional nanofillers such as silver nanoparticles (AgNPs), zinc oxide nanoparticles (ZnO NPs), titanium dioxide nanoparticles (TiO_2_ NPs), and bioactive glass (BAG), whose effects depend on composition and formulation. Contact-active systems incorporate immobilized antibacterial components within the cured resin network and provide surface-associated antibacterial activity; representative examples include 12-methacryloyloxydodecylpyridinium bromide (MDPB), dimethylaminohexadecyl methacrylate (DMAHDM), and other quaternary ammonium methacrylates (QAMs), quaternary ammonium polyethylenimine nanoparticles (QPEI NPs), and methacrylated chitosan. Multifunctional systems combine antibacterial activity with complementary functions such as remineralization, protein repellence, and ion recharge, as represented by DMAHDM + nanoparticles of amorphous calcium phosphate (NACP), 2-methacryloyloxyethyl phosphorylcholine (MPC) + DMAHDM, and rechargeable NACP systems. Because these systems incorporate release-based and/or contact-active antibacterial components, they may also retain the corresponding advantages and limitations of the incorporated antibacterial strategy. Overall material design requires balancing antibacterial efficacy with polymerization, mechanical integrity, aging durability, and biocompatibility. Colors distinguish the major design strategies: blue indicates soluble agents and functional nanofillers, green indicates contact-active systems, orange indicates multifunctional systems, and purple indicates the overall design balance. Arrows and dashed lines schematically indicate release or functional relationships among the corresponding components. AgNPs, silver nanoparticles; BAG, bioactive glass; CHX, chlorhexidine; DMAHDM, dimethylaminohexadecyl methacrylate; MDPB, 12-methacryloyloxydodecylpyridinium bromide; MPC, 2-methacryloyloxyethyl phosphorylcholine; NACP, nanoparticles of amorphous calcium phosphate; QAMs, quaternary ammonium methacrylates; QPEI NPs, quaternary ammonium polyethylenimine nanoparticles; TiO_2_ NPs, titanium dioxide nanoparticles; ZnO NPs, zinc oxide nanoparticles. (Image generated using ChatGPT (OpenAI; GPT-5.6 Sol) and edited by the authors).

Original research articles, systematic reviews, and relevant narrative reviews published in English were considered. Priority was given to publications that evaluated antibacterial activity together with polymerization behavior, mechanical properties, water-related degradation, biocompatibility, or long-term performance. Recent studies were considered alongside earlier publications representing foundational developments or remaining directly relevant to the scope of this review, including studies on MDPB, QAMs, antibacterial nanoparticles, and rechargeable calcium phosphate systems. Publications unrelated to resin-based dental materials, reports lacking sufficient methodological information, and studies focused solely on nonrestorative applications were excluded. Because this article is a narrative review, publication selection and evidence synthesis were qualitative and were not conducted according to a formal systematic-review protocol. To illustrate publication activity in this field, a focused PubMed search was additionally performed, and records relevant to antibacterial resin-based dental restorative composites were retained after manual screening. The annual distribution of these publications is shown in Figure 1. The principal polymer-composite design strategies discussed in this review are summarized schematically in Figure 2 and comparatively in Table 1, providing an overview for the subsequent sections.

## 3. Leachable Antibacterial Agents and Nanofillers

**Figure 3:**
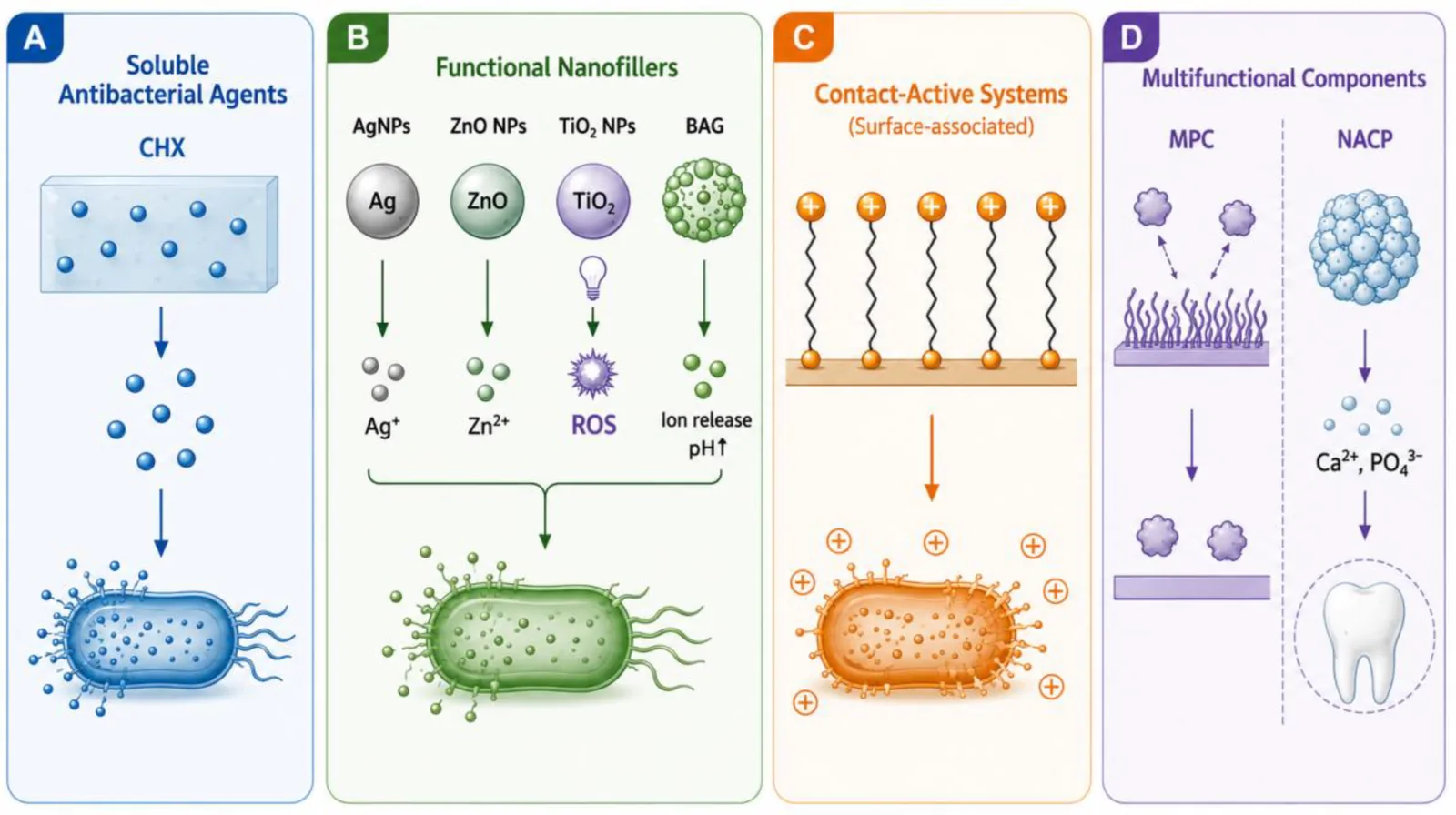
Representative antibacterial and complementary mechanisms in dental resin composites. (**A**) Chlorhexidine (CHX) represents a soluble antibacterial agent that can diffuse from the resin matrix and exert antibacterial effects after release. (**B**) Functional nanofillers exhibit composition-dependent mechanisms: silver nanoparticles (AgNPs) can release Ag^+^, zinc oxide nanoparticles (ZnO NPs) can release Zn^2+^, titanium dioxide nanoparticles (TiO_2_ NPs) can generate reactive oxygen species (ROS) under appropriate illumination, and bioactive glass (BAG) can undergo ion release and contribute to local pH modulation. (**C**) Contact-active systems contain immobilized cationic groups that provide surface-associated antibacterial activity through interaction with bacterial cell envelopes. (**D**) Multifunctional components provide complementary functions: 2-methacryloyloxyethyl phosphorylcholine (MPC) reduces protein adsorption at the material surface, whereas nanoparticles of amorphous calcium phosphate (NACP) release Ca^2+^ and PO_4_^3−^ ions to support remineralization and, in appropriately designed systems, ion recharge. AgNPs, silver nanoparticles; BAG, bioactive glass; CHX, chlorhexidine; MPC, 2-methacryloyloxyethyl phosphorylcholine; NACP, nanoparticles of amorphous calcium phosphate; ROS, reactive oxygen species; TiO_2_ NPs, titanium dioxide nanoparticles; ZnO NPs, zinc oxide nanoparticles. (Image generated using ChatGPT (OpenAI; GPT-5.6 Sol) and edited by the authors).

Antibacterial materials discussed in this section include soluble molecular agents and functional nanofillers, but their release behavior and antibacterial mechanisms differ substantially. For soluble agents such as CHX, activity depends mainly on water-mediated dissolution and diffusion from the polymer network, and progressive release may lead to reservoir depletion and changes in material properties [9,10,11,12]. Antibacterial nanofillers, in contrast, exhibit particle- and formulation-dependent mechanisms and are discussed separately below. The major mechanisms of the representative antibacterial components discussed in this review are summarized in Figure 3.

### 3.1. Chlorhexidine and Other Releasable Agents

CHX has been widely investigated as a releasable antibacterial agent in dental resin composites [9,10,11,12]. Direct incorporation into the resin matrix can provide an early antibacterial effect, but freely dispersed CHX may be released rapidly and leave voids within the material. Increasing the CHX content may enhance release and antibacterial activity, but it can also increase water sorption and compromise mechanical properties [9].

Carrier-based delivery has been developed to improve CHX dispersion and regulate its release. Mesoporous silica particles have a high internal surface area and can retain CHX within their pores before gradual diffusion into the surrounding medium. CHX-loaded mesoporous silica composites have shown antibacterial activity against *Streptococcus mutans* (*S. mutans*), although their release behavior and mechanical properties remain dependent on carrier concentration and filler loading [10,11]. Montmorillonite has also been evaluated as a CHX carrier. A recent study reported that composites containing CHX-loaded montmorillonite maintained antibacterial activity after one year, while CHX release, degree of conversion, sorption, solubility, flexural strength, and elastic modulus varied according to the filler formulation [12].

Carrier particles may reduce burst release and extend antibacterial activity, but the amount of releasable CHX remains finite. Water uptake facilitates CHX diffusion from the resin matrix, while sustained release may be accompanied by changes in sorption, solubility, and material properties [9,12]. The value of CHX-releasing systems therefore depends on maintaining an effective local concentration without causing excessive sorption, solubility, or loss of mechanical integrity.

### 3.2. Silver, Zinc Oxide, and Titanium Dioxide Nanoparticles

Antibacterial nanofillers can exert antimicrobial effects through mechanisms that vary with particle composition and formulation. In silver nanoparticle (AgNP)-containing systems, antibacterial activity is not attributable solely to released Ag^+^ ions. The high surface area and surface reactivity of AgNPs can promote direct interactions with bacterial membranes, while released Ag^+^ ions can interfere with membrane-associated proteins, enzymes, and intracellular components. These combined effects may increase membrane permeability and disrupt cellular metabolism [26,27,28]. AgNP-containing resin materials have reduced bacterial growth and biofilm formation, but their performance depends on particle size, concentration, surface modification, and dispersion [26,27,28].

Agglomeration is an important limitation of AgNP-containing composites. Poorly dispersed particles may impair light transmission, produce nonuniform ion release, and create defects within the cured material. Immobilization on halloysite nanotubes and the use of compatible surface coatings have therefore been investigated to improve particle distribution within the resin matrix [27]. A 2024 study applied AgNPs to the surface of a bis-acrylic temporary restorative resin and reported reduced biofilm formation by *Streptococcus sanguinis* and *Actinomyces naeslundii*. The study also detected concentration-dependent effects on human gingival fibroblast viability, indicating that antibacterial performance and cytocompatibility must be evaluated together [28].

Their incorporation into dental resin materials has produced antibacterial activity, although their effects on material properties depend on particle concentration, dispersion, and the type of resin system. Nanoparticle incorporation may also influence bonding performance to enamel and dentin [29]. More recent work has examined photoactivated zinc oxide nanoparticles (ZnO NPs) in orthodontic resin composites and reported antibiofilm and antidemineralization effects without a significant reduction in the selected physical and mechanical outcomes at the tested concentration [30]. ZnO NPs can exert antibacterial effects through multiple mechanisms, including direct particle–bacterial surface interactions, Zn^2+^ release, and reactive oxygen species (ROS) generation. Surface interactions may compromise bacterial membrane integrity, while released Zn^2+^ and oxidative stress can interfere with cellular proteins and metabolic processes [31]. Importantly, photocatalytic ROS generation by photoactivated ZnO is illumination-dependent and therefore should not be considered continuously active in the oral cavity. Antibacterial effects occurring outside periods of suitable illumination may instead involve non-photocatalytic mechanisms such as particle–surface interactions and Zn^2+^ release [31]. Nevertheless, most evidence remains preclinical, and direct comparison among formulations is limited by differences in particle characteristics and testing methods.

Titanium dioxide nanoparticles (TiO_2_ NPs) produce antibacterial activity mainly through photocatalytic generation of ROS. Conventional TiO_2_ requires ultraviolet activation, which restricts its clinical applicability [32]. Nitrogen-doped TiO_2_ has therefore been developed to extend photocatalytic activity toward the visible-light range and improve its suitability as an esthetic antimicrobial filler in dental polymers [33]. Importantly, photocatalytic antibacterial activity should not be assumed to operate continuously in the oral cavity, because ROS generation depends on exposure to light of sufficient wavelength and intensity. Conventional TiO_2_ would therefore be expected to show limited photocatalytic activity under routine intraoral conditions, whereas visible-light-responsive or doped TiO_2_ may become active only during periods of suitable illumination. Any antibacterial persistence after illumination should be distinguished from continuous photocatalysis itself [32,33]. The magnitude and duration of these effects under clinically relevant intraoral illumination and after prolonged aging remain to be established.

Other nanostructured fillers have been developed to improve compatibility between the antibacterial phase and the polymer matrix. In one recent approach, cerium oxide nanoparticles were deposited onto mesoporous silica particles and incorporated into a resin composite. The particles were surface-functionalized with a silane coupling agent to improve interfacial compatibility with the methacrylate resin matrix. Silanization promotes coupling between the inorganic particle surface and the organic resin phase, thereby improving filler–matrix interaction and helping to limit the loss of mechanical performance associated with nanoparticle incorporation. In this study, silane-treated formulations showed higher flexural strength and depth of cure than the corresponding untreated formulations, and most satisfied the International Organization for Standardization (ISO) 4049 flexural-strength requirement [34]. This finding illustrates the importance of carrier architecture and interfacial treatment in limiting the adverse material effects of antibacterial nanoparticles.

### 3.3. Bioactive and Ion-Releasing Glass Fillers

Bioactive glass (BAG) fillers differ from conventional antibacterial nanoparticles because their biological effects arise primarily from glass dissolution and ion exchange. Depending on their composition, BAG particles can release Ca^2+^, PO_4_^3−^, Na^+^, and other ions. These reactions can increase the local pH, promote apatite precipitation, and create conditions that are less favorable for acidogenic biofilms [18,35,36]. Their effects are influenced by glass composition, particle size, filler loading, surface treatment, and water access to the reactive glass phase. The antibacterial effect of conventional BAG is therefore mainly associated with glass dissolution, ion exchange, and the resulting increase in local pH rather than a direct contact-killing mechanism. This differs from NACP, whose Ca^2+^ and PO_4_^3−^ release is primarily intended to increase mineral availability and support remineralization and acid buffering, without necessarily producing a direct antibacterial effect.

The amount of BAG incorporated into the composite is a critical design variable. BAG loading must be carefully optimized, because low filler content may limit ion release, whereas higher loadings can adversely affect water-related and mechanical properties [35,36,37]. A 2024 study evaluated composites containing 1.9, 3.8, and 7.7 vol% 45S5 BAG. These materials reduced the viability and thickness of *S. mutans* biofilms and promoted calcium phosphate deposition after appropriate preconditioning. However, the initial extracts and direct contact with freshly cured BAG-containing specimens reduced cellular metabolic activity, showing that biological responses depend on both BAG concentration and material conditioning [36].

Polymer-matrix modification has also been investigated to offset the material limitations associated with reactive glass fillers. A 2024 study combined 45S5 BAG with polyhedral oligomeric silsesquioxane (POSS) and found that an appropriate POSS concentration improved degree of conversion and mechanical properties while preserving acid neutralization, Ca^2+^ and PO_4_^3−^ release, and apatite formation. Higher POSS concentrations caused agglomeration and reduced performance [37]. This study did not directly assess antibacterial activity, but it demonstrates how polymer-network design can improve the balance between bioactivity and structural performance.

BAG can also be doped with therapeutic ions to introduce or strengthen antibacterial activity. Copper-doped mesoporous BAG nanospheres have shown antibacterial effects when incorporated into dental composites while retaining ion release and mineralization-related functions [38]. Silver-containing BAG systems similarly combine antibacterial ion release with apatite-forming potential [39]. Accordingly, conventional BAG and ion-doped BAG should be distinguished: the former acts mainly through dissolution-driven changes in pH and ionic conditions, whereas incorporation of antibacterial ions such as Cu or Ag can introduce additional direct antimicrobial effects. These findings highlight the need to optimize the type and concentration of ion-doped bioactive glass fillers when balancing antibacterial activity with overall material performance [38,39]. The composition and concentration of doped glass fillers must therefore be carefully controlled.

Overall, soluble leachable agents and antibacterial nanofillers can act beyond direct bacterial contact, although their release behavior, antibacterial mechanisms, and interactions with the polymer network differ. CHX carriers may extend drug release, whereas AgNPs, ZnO NPs, TiO_2_ NPs, and BAG fillers provide ion-mediated, photocatalytic, or pH-related effects. Their clinical translation requires sustained antibacterial activity after aging while maintaining acceptable overall material performance and biological safety.

## 4. Contact-Active Antibacterial Polymer Networks

Contact-active antibacterial materials contain cationic groups that are immobilized within the cured polymer network. Unlike leachable systems, their activity does not depend on continuous diffusion of an antibacterial agent into the surrounding medium. The antibacterial effect occurs mainly at the material surface, where accessible positively charged groups interact electrostatically with negatively charged components of the bacterial cell envelope. This interaction can disrupt membrane organization and permeability, leading to loss of membrane integrity and bacterial cell death. For QAMs containing hydrophobic alkyl chains, interaction of the alkyl moiety with the lipid membrane can further enhance membrane disruption. Performance is influenced by monomer structure, concentration, surface charge density, polymerization, and the accessibility of active groups after aging and biofilm formation [8,15,16].

### 4.1. MDPB-Based Systems

MDPB was one of the first polymerizable antibacterial monomers developed for dental resins. Its methacrylate group participates in copolymerization, whereas the positively charged quaternary pyridinium group contributes to antibacterial activity through electrostatic interaction with the negatively charged bacterial cell envelope and subsequent disruption of membrane integrity. Early studies demonstrated that MDPB could be incorporated into resin composites and retain antibacterial activity after curing [13,21,22,23]. The unpolymerized monomer shows bactericidal activity in solution, while polymerized MDPB provides surface-associated antibacterial effects [22,23,40].

The concentration of MDPB must be controlled because its incorporation can affect resin polymerization. Early work showed that increasing the MDPB content influenced curing behavior, although suitable formulations could retain clinically relevant polymerization characteristics [21]. MDPB-containing composites have also been evaluated for water sorption and color stability, with the magnitude of change depending on the incorporated concentration [41]. In addition to restorative composites, MDPB has been incorporated into dentin primers to provide antibacterial activity before polymerization and to reduce viable bacteria remaining on treated dentin [42].

The activity of polymerized MDPB is concentrated at the resin surface and therefore depends on bacterial contact with accessible pyridinium groups. Experimental studies support the antibacterial activity of MDPB-containing dental materials [40,41,42]; however, these studies do not provide direct evidence of long-term clinical prevention of secondary caries. Its major contribution was to establish that an antibacterial monomer could be immobilized within a dental polymer rather than continuously released.

### 4.2. Other QAMs

The development of QAMs has expanded from MDPB to monomers with different alkyl-chain lengths, charge densities, and numbers of polymerizable groups. DMAHDM is among the most extensively investigated QAMs for experimental dental materials. Its methacrylate group enables incorporation into the polymer network, while the quaternary ammonium group contributes to surface-associated antibacterial activity [14,15,16]. Increasing the DMAHDM concentration can increase surface charge density and enhance antibiofilm activity, although the acceptable concentration depends on the resin formulation and intended application [15,43].

Alkyl-chain length is an important structural variable in QAM design. In experimental calcium phosphate nanocomposites containing QAMs with alkyl-chain lengths from 3 to 18 carbons, antibacterial activity increased with chain length and reached its maximum at a chain length of 16 before decreasing at 18 carbons. The C16 formulation produced approximately a 10-fold reduction in biofilm metabolic activity and acid production compared with the control, without adversely affecting the measured mechanical properties [16]. DMAHDM-containing materials have reduced biofilm colony-forming units, metabolic activity, lactic acid production, and extracellular polysaccharide production in laboratory models [14,43,44]. In a composite containing 20% NACP, increasing DMAHDM from 3% to 5% enhanced antibacterial activity without significantly reducing flexural strength or elastic modulus under the tested conditions [43].

The number of polymerizable groups provides another means of modifying network structure. Experimental quaternary ammonium monomers containing two or four methacrylate groups were evaluated at 1, 4, and 7 mass%, with antibacterial activity increasing as monomer concentration increased. At 4 mass%, the tetrafunctional TMH-DB-containing resin showed a higher storage modulus and crosslink density than the corresponding dimethacrylate MAE-DB formulation, while their glass-transition temperatures were similar [45]. A tetrafunctional methacrylate quaternary ammonium monomer was also developed to increase integration within the crosslinked network. At an appropriate concentration, the resulting experimental resin showed antibacterial activity against *S. mutans*, increased crosslink density, and no significant reduction in degree of conversion compared with the unmodified resin [46].

Hydrolytic stability is another concern because ester-containing methacrylates may degrade under aqueous and acidic conditions. Quaternary ammonium-containing methacrylamides have therefore been investigated as hydrolysis-resistant alternatives. A 2025 study synthesized pyridinium- and *N*-alkyl ammonium-containing methacrylamides with activity against *S. mutans* and *Enterococcus faecalis*. Their antibacterial activity varied with alkyl-chain length and intermolecular interactions. The synthesized monomers and polymers showed resistance to acidic hydrolysis, but their degree of conversion was generally lower than that of MDPB at a 30 s irradiation time [47]. The study evaluated experimental resin-adhesive formulations rather than filled restorative composites, so further testing of filler compatibility, aging, and restorative performance is required.

Contact-active materials should be evaluated after aging rather than only immediately after curing. Water storage can alter polymer structure and surface properties, while accumulated proteins and biofilm matrix may reduce direct access to immobilized cationic groups. Surface wear and abrasion may also alter the density and accessibility of immobilized cationic groups at the exposed material surface, potentially modifying contact-active antibacterial activity during clinical service. Therefore, the durability of contact-active systems should be assessed under combined aging and mechanical-wear conditions rather than by water storage alone. Selected DMAHDM-containing formulations have retained antibacterial activity after prolonged water aging [48]. Nevertheless, differences in resin composition, QAM concentration, aging conditions, and microbiological methods limit direct comparison among studies.

### 4.3. Polymeric and Biopolymeric Antibacterial Agents

Quaternary ammonium polyethylenimine nanoparticles (QPEI NPs) provide antibacterial activity through a densely cationic polymeric structure. Because the quaternary ammonium groups are incorporated within crosslinked particles, the antibacterial component can remain largely immobilized after addition to the resin. Experimental composites containing QPEI NPs have reduced *S. mutans* viability, bacterial adherence, and biofilm formation, although the magnitude of the effect depends on particle concentration and formulation [49,50].

QPEI NP incorporation can also influence the physicochemical and mechanical properties of the resin. In bisphenol A-glycidyl methacrylate (Bis-GMA)/triethylene glycol dimethacrylate (TEGDMA) composites containing 0.5–2 wt.% QPEI NPs, degree of conversion, polymerization shrinkage, water sorption, elastic modulus, and hardness were not significantly impaired, whereas solubility increased and flexural strength and impact resistance decreased as the nanoparticle content increased [51]. Cytocompatibility and bacterial adherence have also been shown to vary with QPEI NP concentration [50]. Careful control of particle loading and dispersion is therefore required to achieve antibacterial activity without unacceptable deterioration of material performance.

Chitosan is a naturally derived cationic polysaccharide with antibacterial and carrier properties. Its positively charged amino groups can interact electrostatically with negatively charged microbial surfaces, altering membrane permeability and compromising membrane integrity, thereby contributing to its antimicrobial activity [52,53]. Its reactive functional groups also permit further chemical modification. Native chitosan has limited compatibility with hydrophobic methacrylate matrices, and methacrylation has been used to improve its incorporation into conventional methacrylate-based resin networks [52,53,54].

Experimental composites and adhesives containing methacrylated chitosan have shown reduced biofilm formation, although their mechanical behavior varies with concentration and resin formulation [54]. A 2025 systematic review concluded that chitosan-modified resin composites generally showed antibacterial potential, but the available studies were heterogeneous in chitosan form, concentration, material composition, test organisms, and outcome measures [52]. Current evidence remains predominantly laboratory-based, and long-term aging, optical stability, biocompatibility, and clinical effectiveness require further investigation.

Contact-active polymer networks provide antibacterial activity without relying on a finite releasable reservoir. Their effectiveness depends on the density and accessibility of immobilized active groups, whereas their suitability as restorative materials also requires adequate polymerization, mechanical stability, and biological safety. MDPB and DMAHDM remain the most established monomer-based systems, while multifunctional QAMs, hydrolysis-resistant methacrylamides, QPEI NPs, and methacrylated chitosan represent developing approaches for improving antibacterial activity and integration within dental polymer networks. The principal antibacterial agents and functional fillers discussed above are summarized in Table 2.

## 5. Multifunctional Antibacterial Resin Composites

Multifunctional resin composites combine antibacterial activity with additional functions that address other aspects of the caries process. These functions include calcium and phosphate ion release, remineralization, protein repellence, acid neutralization, and ion recharge. The rationale is that antibacterial activity alone does not prevent protein adsorption or restore mineral lost during repeated acidic challenges. However, combining several active components increases formulation complexity and may influence polymerization and overall material performance [6,17]. Potential interactions among antibacterial monomers, protein-repellent comonomers, and reactive fillers may affect resin viscosity, polymerization kinetics, filler dispersion, filler–matrix compatibility, water uptake, and ion release. Therefore, the performance of multifunctional formulations cannot be predicted solely from the properties of individual components, and compatibility should be evaluated for each specific combination.

### 5.1. Antibacterial and Remineralizing Systems

NACP has been widely investigated as a remineralizing filler in dental resin materials, and the released Ca^2+^ and PO_4_^3−^ ions can increase local mineral availability and promote remineralization of demineralized enamel [55]. NACP-containing composites release Ca^2+^ and PO_4_^3−^ ions after exposure to aqueous media, with release increasing under acidic conditions [56,57]. In a human in situ model, an NACP nanocomposite released Ca and P ions and reduced enamel mineral loss adjacent to restorations compared with a glass-filled control, although it did not significantly reduce biofilm colony-forming units [57]. This distinction indicates that the caries-inhibitory effect of NACP may arise primarily from ion release and mineral protection rather than direct bacterial killing.

NACP has therefore been combined with contact-active antibacterial monomers. DMAHDM and NACP represent one of the most extensively investigated combinations. DMAHDM reduces biofilm viability and acid production, whereas NACP supplies Ca^2+^ and PO_4_^3−^ ions. In experimental composites, selected DMAHDM/NACP formulations reduced *S. mutans* biofilm activity and enamel demineralization at restoration margins without significantly reducing the measured flexural properties [43,44]. These results were obtained under laboratory conditions and should not be interpreted as evidence of clinical prevention of secondary caries.

The proportions of the antibacterial monomer and remineralizing filler require careful control. Increasing DMAHDM concentration can strengthen antibacterial activity, while increasing NACP loading generally increases ion release. However, changes in either component can affect resin viscosity, filler packing, polymerization, flow, and mechanical properties [43,58]. The most effective formulation is therefore not necessarily the one containing the highest concentration of each active component.

BAG has also been used to combine mineralization with antibiofilm activity. In a 2024 study, urethane dimethacrylate (UDMA)/TEGDMA composites containing 1.9, 3.8, or 7.7 vol% 45S5 BAG promoted calcium phosphate deposition and reduced the viability and thickness of *S. mutans* biofilms. However, extracts from freshly cured BAG-containing specimens and direct contact with these specimens reduced cellular metabolic activity. Preconditioning improved cellular responses, particularly for the 7.7 vol% formulation [36]. These findings show that mineralization, antibiofilm activity, and cytocompatibility should be evaluated together when reactive fillers are incorporated into resin composites.

### 5.2. Protein-Repellent and Antibacterial Systems

Protein adsorption contributes to the formation of a conditioning layer on resin surfaces and precedes bacterial attachment. Protein-repellent monomers have therefore been combined with contact-active antibacterial monomers to reduce surface fouling and bacterial accumulation [17]. The most extensively studied protein-repellent monomer is 2-methacryloyloxyethyl phosphorylcholine (MPC). Its phosphorylcholine group promotes the formation of a highly hydrated surface, thereby limiting protein adsorption [17,59]. More broadly, surface engineering provides an additional strategy for limiting microbial adhesion to dental materials. Highly hydrophilic surfaces can maintain a strongly hydrated interfacial layer that reduces nonspecific protein adsorption and subsequent bacterial attachment, whereas superhydrophobic surfaces aim to reduce microbial adhesion through low surface energy and surface topography [60]. The effectiveness of these surface properties in the oral environment may nevertheless be modified by salivary conditioning, biofilm formation, and changes in surface roughness or topography during clinical service [60]. In an experimental dental composite, increasing MPC content up to 3 wt% reduced protein adsorption, early bacterial attachment, and biofilm growth without significantly impairing flexural properties. In contrast, formulations containing 4.5 or 6 wt% MPC showed reduced mechanical properties [59]. Thus, the protein-repellent effect is concentration-dependent and must be balanced against changes in the polymer network.

Because MPC is not primarily bactericidal, it has been combined with DMAHDM. A composite containing 3 wt% MPC and 1.5 wt% DMAHDM showed lower protein adsorption, biofilm colony-forming units, metabolic activity, and lactic acid production than the control and the corresponding single-agent formulations [61]. The combined formulation maintained flexural properties comparable to the commercial control under the tested conditions. These findings support a complementary mechanism in which MPC reduces protein adsorption and bacterial attachment, while DMAHDM acts against bacteria reaching the material surface.

The durability of this combination has also been examined. A composite containing 3 wt% MPC and 1.5 wt% DMAHDM retained protein-repellent and antibacterial activity after water storage for up to 180 days, while maintaining load-bearing properties comparable to those of the commercial control [48]. In a separate bonding-agent study, MPC- and DMAHDM-containing primer and adhesive retained protein-repellent and antibacterial effects, as well as dentin bond strength, after six months of water aging [62]. These studies demonstrate laboratory durability, but long-term clinical effectiveness has not yet been established.

### 5.3. Rechargeable and pH-Responsive Systems

Conventional calcium- and phosphate-ion-releasing materials may be limited by depletion of their initial ion reservoir. Rechargeable systems were developed to absorb Ca^2+^ and PO_4_^3−^ from an external solution after the original release has diminished and subsequently release the ions again. Recharge and re-release depend on NACP loading and the affinity of the resin matrix for calcium and phosphate ions [63,64]. Importantly, the presence of NACP alone does not necessarily confer effective rechargeability; repeated ion uptake and re-release depend on both NACP loading and the chemical affinity of the surrounding resin matrix for calcium and phosphate ions.

Rechargeable NACP composites have shown repeated Ca and P ion recharge and re-release without a progressive reduction in ion output. In one study, different resin matrices were compared, and the formulation containing pyromellitic glycerol dimethacrylate showed the greatest recharge capability. After six recharge and re-release cycles, the recharged composites continued to release Ca and P ions for 42 days without further recharge [63]. Their flexural strength was approximately three times that of the tested resin-modified glass ionomer controls, while the elastic moduli were similar.

Antibacterial activity has also been incorporated into rechargeable composites. A formulation containing 3 wt% DMAHDM and 20 wt% NACP underwent 12 recharge and re-release cycles over six months without a significant reduction in ion release. It also reduced biofilm colony-forming units by approximately four orders of magnitude and decreased biofilm acid production compared with the control [25]. These findings demonstrate that renewable ion delivery and contact-active antibacterial activity can be combined within the same resin composite.

Rechargeable systems have also been developed for sealants and adhesives. In an experimental sealant, increasing NACP content from 0 to 30 wt% increased Ca^2+^ and PO_4_^3−^ release and rechargeability. The 20 wt% formulation provided a suitable balance of ion release, flexural properties, and flow, whereas 30 wt% NACP significantly reduced flowability [58]. In experimental adhesives, increasing NACP loading increased initial ion release and recharge capacity without significantly reducing dentin shear bond strength. Recharged specimens maintained ion re-release through repeated cycles and continued releasing ions for several weeks after the final recharge [64].

NACP-containing materials also show passive pH-responsive behavior because acidic conditions increase filler dissolution and Ca^2+^ and PO_4_^3−^ release. In an in vitro study of experimental sealants, formulations containing 20 wt% NACP, with or without DMAHDM, reduced mineral loss in artificially demineralized human enamel during pH cycling [65]. The study supports pH-responsive ion release and protection against demineralization, but it did not directly assess biofilm inhibition. These systems are therefore better described as passive pH-responsive materials rather than externally controlled smart-release platforms.

Multifunctional composites can provide broader activity than materials based on a single mechanism, but their performance depends on interactions among the resin matrix, antibacterial monomers, protein-repellent components, and reactive fillers. Each intended function should be confirmed after aging and should not be assumed from the presence of an active ingredient alone. Current evidence supports the laboratory feasibility of antibacterial, remineralizing, protein-repellent, and rechargeable systems, but clinical studies are required to determine whether these properties lead to sustained prevention of secondary caries. Representative multifunctional antibacterial systems and their principal functions, reported benefits, and design limitations are summarized in Table 3.

## 6. Structure–Property Relationships and Translational Challenges

**Figure 4:**
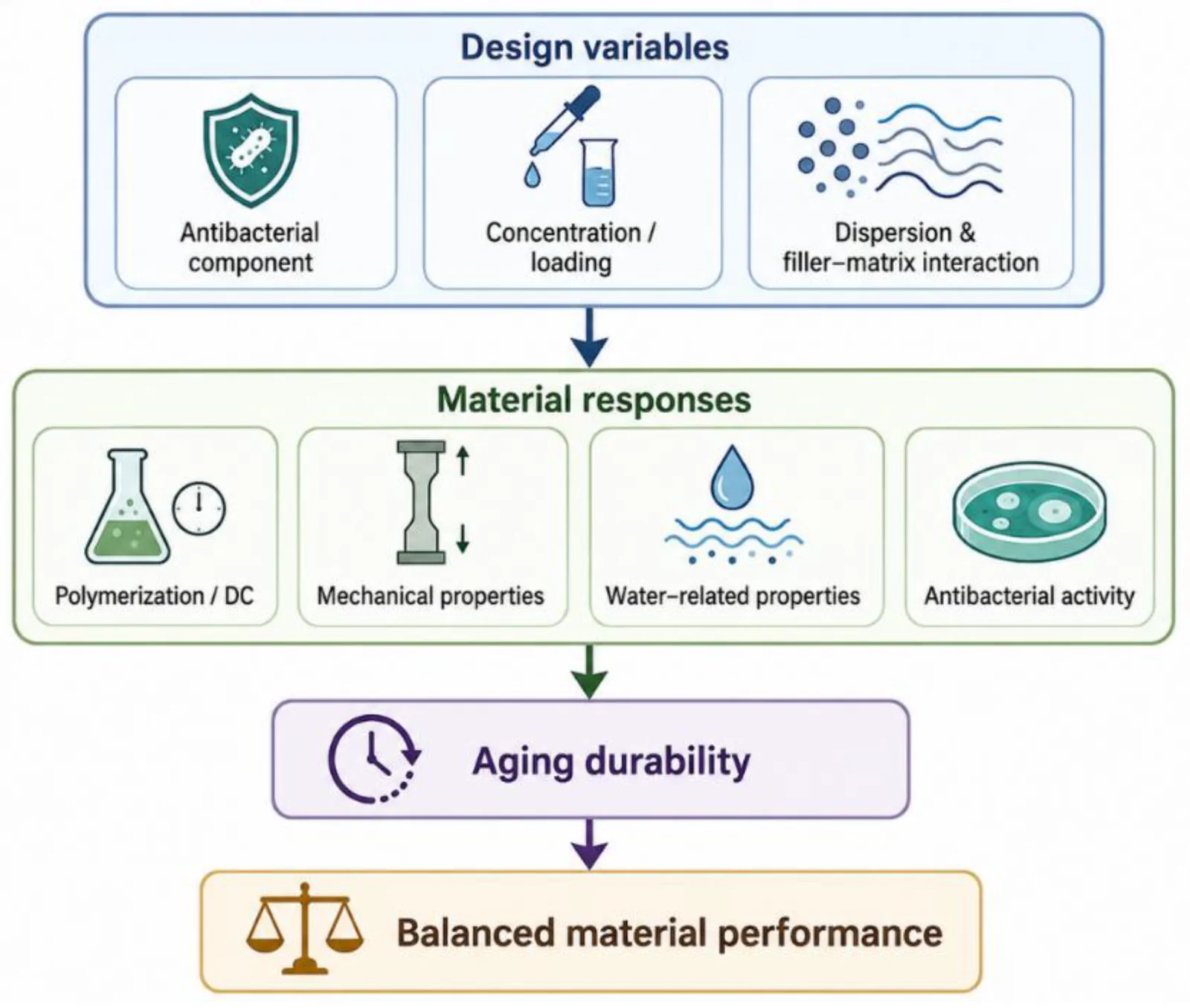
Structure–property relationships and aging considerations for antibacterial dental resin composites. Key design variables include the type of antibacterial component, its concentration or filler loading, and the quality of dispersion and filler–matrix interaction. These variables can influence polymerization and degree of conversion, mechanical properties, water-related behavior, and antibacterial activity. Long-term material performance further depends on the durability of these properties during aging. Successful formulation therefore requires balancing antibacterial efficacy with polymerization, mechanical integrity, water-related stability, and aging durability. Colors distinguish the major stages of the framework: blue indicates design variables, green indicates material responses, purple indicates aging durability, and orange indicates balanced material performance. Arrows indicate the sequential relationships from material design to material response, aging durability, and overall performance. DC, degree of conversion. (Image generated using ChatGPT (OpenAI; GPT-5.6 Sol) and edited by the authors).

Antibacterial activity cannot be considered independently of the properties required for a restorative composite. Changes in monomer composition, filler loading, particle surface treatment, and hydrophilicity may affect polymerization, mechanical behavior, water uptake, optical properties, and biological safety. A formulation that performs well in a short-term antibacterial assay may therefore be unsuitable if the active component interferes with polymer-network formation or accelerates degradation during clinical service. Evaluation of antibacterial composites should include both biological efficacy and the material properties that determine restoration longevity, as summarized in Figure 4 [1,6].

### 6.1. Degree of Conversion and Mechanical Properties

Degree of conversion is an important parameter because it influences residual monomer content, network stability, mechanical properties, and biocompatibility. Antibacterial monomers may alter resin viscosity, radical mobility, or polymerization kinetics, whereas antibacterial fillers may affect light transmission through scattering or absorption. These effects depend on the chemical structure, concentration, and compatibility of the incorporated component. MDPB, QAMs, QPEI NPs, and multifunctional fillers have shown concentration-dependent effects on polymerization and mechanical performance [21,43,46,51]. For example, in a composite containing 20 wt% NACP, increasing DMAHDM from 3 to 5 wt% enhanced antibacterial activity without significantly reducing flexural strength or elastic modulus under the tested conditions [43].

Increasing the concentration of a contact-active monomer may improve surface charge density and antibacterial activity, but excessive incorporation can interfere with conversion or network formation. The number and arrangement of polymerizable groups also influence network behavior. Experimental quaternary ammonium monomers containing two or four methacrylate groups were evaluated at 1, 4, and 7 mass%, with antibacterial activity increasing as monomer concentration increased. At 4 mass%, the tetrafunctional TMH-DB-containing resin showed a higher storage modulus and crosslink density than the corresponding dimethacrylate MAE-DB formulation, while their glass-transition temperatures were similar [45]. A related tetrafunctional methacrylate quaternary ammonium monomer also produced antibacterial activity against *S. mutans*, increased crosslink density, and no significant reduction in degree of conversion at an appropriate concentration [46]. The acceptable loading is therefore specific to each resin formulation and cannot be transferred directly among composites, adhesives, sealants, and temporary restorative materials.

Functional fillers introduce similar trade-offs. Reactive particles may replace part of the reinforcing filler fraction, alter filler packing, or affect filler–matrix coupling. Increasing NACP loading can enhance ion release but may reduce flow or alter mechanical properties, whereas BAG concentration can affect conversion, strength, water-related behavior, and cellular responses [36,43,58]. Nanoparticle agglomeration can compromise dispersion and create structural heterogeneity, while inadequate particle distribution may also adversely affect light transmission and curing behavior [27,34,37]. Surface modification can improve dispersion and filler–matrix interaction, but it may also alter water access and ion release.

Antibacterial efficacy should therefore be interpreted together with degree of conversion, flexural strength, elastic modulus, hardness, wear resistance, and optical stability. Measurements performed immediately after curing are insufficient for materials intended to function over several years. Relevant properties should also be evaluated after water storage, thermal cycling, mechanical fatigue, or other aging procedures.

### 6.2. Water Sorption, Aging, and Antibacterial Durability

Water is required for the release of CHX, Ca^2+^, PO_4_^3−^, and therapeutic ions from reactive fillers, but it also contributes to polymer degradation. Water uptake can plasticize the resin matrix, facilitate hydrolysis of ester-containing monomers and silane interfaces, and promote filler dissolution or debonding [1]. CHX-containing and other soluble agent systems may undergo additional mass loss as the active component leaves the material [9,12]. In contrast, the aging behavior of antibacterial nanofillers is more dependent on particle composition, concentration, dispersion, and filler–matrix interactions than on depletion of a soluble reservoir [26,27,28,29,30,32,33,34]. Contact-active systems avoid depletion of the antibacterial reservoir, but aging may still alter the polymer network and the accessibility of cationic groups at the surface [8,48].

Short-term antibacterial findings may overestimate durability. Soluble-agent formulations can show strong initial inhibition that decreases as the available reservoir is depleted, whereas antibacterial nanofillers may retain activity through ion-mediated, surface-associated, or photocatalytic mechanisms, depending on their composition and formulation [26,27,28,29,30,32,33]. Their effects on polymerization and material properties are likewise formulation-dependent; appropriate particle concentration and dispersion may preserve material performance, whereas agglomeration or unfavorable particle distribution may impair light transmission, curing behavior, and structural integrity [27,29,30,34]. Several formulations have demonstrated extended functional durability under laboratory aging conditions. CHX-loaded montmorillonite composites maintained antibacterial effects after one year, although CHX release and material properties depended on filler formulation [12]. MPC/DMAHDM composites retained protein-repellent and antibacterial activity after 180 days of water storage [48]. In addition, a rechargeable composite containing 3 wt% DMAHDM and 20 wt% NACP maintained Ca and P ion release through 12 recharge/re-release cycles over six months [25]. These findings demonstrate the feasibility of extended activity but should not be generalized to all materials within the same category.

Aging protocols should clearly specify storage medium, temperature, duration, specimen geometry, surface treatment, and the timing of biofilm exposure. Water storage alone does not reproduce the combined effects of salivary enzymes, thermal changes, dietary acids, mechanical loading, toothbrushing, and stresses at restoration margins.

### 6.3. Biocompatibility and Resistance Concerns

Biocompatibility depends on the identity, concentration, mobility, and formulation of the antibacterial component. Soluble antibacterial agents may diffuse from the polymer matrix, whereas ion-releasing fillers may expose surrounding tissues to released ions. Residual monomers may also be released when polymerization is incomplete. Increasing antibacterial loading may improve microbial inhibition but can also increase cytotoxicity or adversely affect cellular responses, depending on the incorporated component and formulation. Concentration-dependent effects have been reported for QAMs, QPEI NPs, chitosan-modified composites, and BAG-containing formulations [36,47,50,52].

Biological testing should distinguish among uncured monomers, freshly polymerized materials, material extracts, and aged specimens. Cytotoxicity measured using an isolated monomer at a high concentration does not directly predict the response to a cured composite. Therefore, biological safety should be evaluated not only immediately after polymerization but also after appropriate aging periods. Evaluation should use relevant oral cell types and include both direct-contact and extract-based methods under exposure conditions appropriate to the intended application.

Possible microbial adaptation also requires investigation. Soluble leachable agents may expose bacteria to declining concentrations as the available reservoir is depleted, whereas contact-active materials exert a localized surface challenge. Nanoparticle-containing systems should be considered separately because their antibacterial activity may involve ion-mediated, surface-associated, or photocatalytic mechanisms rather than depletion of a freely soluble antibacterial reservoir. However, available evidence is insufficient to determine whether long-term exposure to most antibacterial dental polymers promotes stable resistance or cross-resistance [8]. Resistance should therefore be regarded as an unresolved safety issue rather than an established consequence. Future studies should examine repeated sublethal exposure, changes in antimicrobial susceptibility, biofilm composition, and possible effects on the oral microbiome.

### 6.4. Clinical Translation Gap and Future Directions

Most evidence on experimental antibacterial resin composites remains preclinical. A recent scoping review included 53 studies published during the preceding five years and found only in vitro, in situ, and animal investigations, with no clinical trials. DMAHDM was the most frequently studied antibacterial agent, while *S. mutans* and colony-forming unit counting were the most commonly used microbiological model and outcome, respectively [66]. These findings confirm that the current evidence base is dominated by laboratory testing and simplified microbiological outcomes.

Monospecies *S. mutans* models are useful for initial screening but do not reproduce the microbial diversity, salivary conditioning, pH fluctuations, extracellular matrix, and mechanical challenges present around clinical restorations. Accordingly, reductions in bacterial counts on polished specimens should not be interpreted as equivalent to improved clinical performance.

Clinical findings from commercially available bioactive resin materials also illustrate the difficulty of translating laboratory bioactivity into improved outcomes. A systematic review and meta-analysis of 10 randomized clinical trials involving 411 participants found no significant differences between bioactive and conventional resin composites in secondary caries or retention loss during follow-up periods ranging from one to eight years [67]. The evaluated materials included ACTIVA BioACTIVE, giomers, and Cention N. These findings apply to the commercial materials included in that review and should not be extrapolated to experimental QAM-, MPC-, or NACP-based composites without comparable clinical evaluation. Several factors may help explain the lack of demonstrated clinical superiority. Secondary caries is multifactorial and is strongly influenced by patient-related caries risk, oral hygiene, dietary exposure, restoration quality, and marginal integrity, which may reduce the measurable contribution of material bioactivity alone. In addition, bioactive effects observed under controlled laboratory conditions may be attenuated clinically by salivary pellicle formation, biofilm accumulation, aging, and intermittent rather than continuous exposure to favorable chemical conditions. Differences in material composition, mechanisms of bioactivity, follow-up duration, and event frequency among clinical trials may also limit the ability to detect modest material-related benefits. These explanations remain hypotheses and require confirmation in adequately powered long-term clinical studies.

Future studies should report material composition, active-agent concentration, aging procedures, and microbiological outcomes in sufficient detail to facilitate interpretation and comparison. Depending on the study objective, clinically relevant models may incorporate multispecies biofilms, salivary conditioning, pH cycling, mechanical loading, or restored-tooth configurations before clinical evaluation. Antibacterial activity against planktonic bacteria should be distinguished from inhibition of established biofilms. Mature biofilms contain an extracellular polymeric matrix, altered bacterial metabolic states, and spatially heterogeneous microbial populations that can reduce susceptibility to antibacterial agents. Therefore, reductions in planktonic bacterial viability should not be interpreted as equivalent to inhibition, disruption, or eradication of mature oral biofilms. Clinical studies should use predefined outcome criteria relevant to restoration performance and secondary caries.

The formulation producing the greatest immediate reduction in bacterial viability may not have the greatest translational potential. Materials providing moderate but durable antibacterial activity, stable polymer networks, controlled ion release, and acceptable biological safety may be more suitable for clinical development. Antibacterial function should therefore be evaluated as one component of overall restorative performance rather than as an isolated endpoint.

## 7. Conclusions

Antibacterial dental resin composites have progressed from release-based formulations to polymer networks integrating contact-active monomers, bioactive fillers, protein-repellent components, and rechargeable ion-delivery systems. Soluble leachable agents can exert antibacterial effects beyond the material surface but may be limited by reservoir depletion and release-related material changes associated with water uptake and component loss. Antibacterial nanofillers, in contrast, exhibit composition- and formulation-dependent ion-mediated, surface-associated, or photocatalytic mechanisms. Contact-active systems based on MDPB, DMAHDM, and other QAMs avoid continuous release, although their effectiveness depends on the density and accessibility of immobilized cationic groups. Multifunctional formulations further integrate antibacterial activity with protein repellence, remineralization, or ion recharge to address multiple stages of the caries process.

Successful development requires balancing antibacterial efficacy with degree of conversion, mechanical and optical properties, hydrolytic stability, and biocompatibility. Because aging, protein adsorption, biofilm accumulation, and acidic challenges may alter both polymer networks and active-component accessibility, short-term results from freshly cured specimens should not be used alone to predict clinical performance. Current evidence supports the laboratory feasibility of antibacterial and multifunctional composites, but clinical validation remains limited. Future development should prioritize durable antibacterial activity, structural integrity, biological safety, and mineral protection through rational control of polymer-network architecture and filler–matrix interactions, followed by validation using clinically relevant aging and biofilm models and long-term clinical studies.

## Figures and Tables

**Table 1:** Comparison of the principal polymer-composite design strategies for antibacterial dental resin composites.

Polymer-Design Strategy	Incorporation Approach	Antibacterial Mechanism	Potential Advantages	Principal Limitations	Representative Systems	References
Soluble leachable-agent systems	Soluble antibacterial agents are physically dispersed within the resin matrix or incorporated into carrier particles. Water penetration enables their dissolution and diffusion from the material.	Released antibacterial compounds act at the composite surface and in the surrounding aqueous environment.	Antibacterial activity can extend beyond the immediate material surface; pronounced initial activity may be achieved; carrier particles may improve dispersion and regulate release.	The releasable reservoir is finite; burst release may cause rapid depletion; component loss may increase porosity, water uptake, sorption, and solubility and may impair mechanical properties.	CHX incorporated directly into the resin; CHX-loaded mesoporous silica or other carrier particles.	[9,10,11,12]
Contact-active systems	Polymerizable antibacterial monomers or immobilized cationic components are incorporated into the cured polymer network and are not intended to diffuse from the material.	Accessible positively charged groups interact with negatively charged bacterial membranes at the material surface.	Reduced dependence on continuous agent release; no finite releasable reservoir; potential for persistent surface-associated antibacterial activity.	Activity requires close bacterial contact and depends on the accessibility and density of surface cationic groups; salivary protein adsorption and biofilm accumulation may mask the active surface; excessive loading may affect polymerization and material properties.	MDPB; DMAHDM and other QAMs; immobilized cationic antibacterial systems.	[8,13,14,15,16,17,21,22,23]
Multifunctional systems	Antibacterial components are combined with protein-repellent monomers, calcium phosphate fillers, acid-neutralizing phases, or rechargeable ion-releasing systems.	Contact-active antibacterial effects are integrated with inhibition of protein adsorption, calcium and phosphate release, acid neutralization, remineralization, or ion rechargeability.	Addresses multiple stages of secondary caries; may simultaneously reduce bacterial viability, bacterial attachment, acid production, and mineral loss; rechargeable systems may permit repeated ion release.	Greater formulation complexity; interactions among monomers and fillers may affect polymerization and overall material performance.	QAM–calcium phosphate systems; antibacterial and protein-repellent formulations; rechargeable calcium phosphate composites.	[1,6,16,17,18,19,20,24,25]

Abbreviations: CHX, chlorhexidine; DMAHDM, dimethylaminohexadecyl methacrylate; MDPB, 12-methacryloyloxydodecylpyridinium bromide; QAMs, quaternary ammonium methacrylates.

**Table 2:** Principal antibacterial agents and functional fillers incorporated into dental resin composites.

Agent or Material Class	Incorporation Type	Principal Antibacterial Mechanism	Reported Advantages	Main Limitations and Design Considerations	References
Chlorhexidine (CHX)	Leachable agent; direct incorporation or carrier-mediated delivery	Diffusion of CHX from the resin matrix; disruption of bacterial cell membranes and inhibition of biofilm activity	Strong initial antibacterial activity; mesoporous silica or other carriers may improve dispersion and prolong release	Finite reservoir; burst release and progressive depletion; increased water sorption, solubility, and void formation; possible deterioration of mechanical properties	[9,10,11,12]
Silver nanoparticles (AgNPs)	Antibacterial nanofiller with ion-releasing and surface-associated activity	Release of Ag^+^ ions and interaction with bacterial membranes, proteins, and intracellular components	Antibacterial and antibiofilm activity in experimental resin formulations	Performance depends on particle concentration, surface modification, and dispersion; agglomeration may impair particle distribution and material performance; cytocompatibility requires consideration at higher concentrations	[26,27,28]
Zinc oxide nanoparticles (ZnO NPs)	Antibacterial nanofiller; photoactivated formulations have also been investigated	Antibacterial activity associated with ZnO nanoparticle incorporation	Antibacterial and antibiofilm activity; potential antidemineralization effects	Performance depends on concentration, dispersion, resin formulation, and activation conditions; incorporation may influence bonding and material properties	[29,30,31]
Titanium dioxide nanoparticles (TiO_2_ NPs)	Photocatalytic nanofiller	Light-activated generation of ROS	Chemically stable, white, and potentially compatible with esthetic resin materials; nitrogen doping may enable visible-light activation	Conventional TiO_2_ generally requires ultraviolet activation; effectiveness under clinically relevant intraoral illumination and after aging remains uncertain	[32,33]
Bioactive glass (BAG) and ion-doped BAG	Reactive, ion-releasing glass filler	Glass dissolution, ion exchange, local pH elevation, and therapeutic-ion release	May combine antibiofilm effects with acid neutralization, apatite formation, and mineralization-related activity	Higher filler loading may adversely affect water-related and mechanical properties; freshly cured BAG-containing materials may show less favorable cellular responses; performance depends on conditioning and glass composition	[18,35,36,37,38,39]
12-Methacryloyloxydodecylpyridinium bromide (MDPB)	Polymerizable contact-active antibacterial monomer	Immobilized quaternary pyridinium groups provide surface-associated antibacterial activity after polymerization through electrostatic interaction with the negatively charged bacterial cell envelope and subsequent disruption of membrane integrity; unpolymerized MDPB is bactericidal in solution	Established proof that an antibacterial monomer can be copolymerized into dental resins; evaluated in composites, primers, and adhesives	Activity is largely surface-confined and requires bacterial contact; concentration may affect curing behavior, water sorption, and color stability; the cited experimental studies do not establish long-term clinical prevention of secondary caries	[13,21,22,23,40,41,42]
Dimethylaminohexadecyl methacrylate and related quaternary ammonium methacrylates (DMAHDM/QAMs)	Polymerizable contact-active antibacterial monomers	Electrostatic interaction and hydrophobic membrane disruption by immobilized cationic groups	Antibacterial activity can be adjusted through alkyl-chain length, charge density, concentration, and number of polymerizable groups; selected formulations retain mechanical properties and activity after water aging	Excessive loading may alter polymerization and mechanical properties; active groups may become less accessible after protein adsorption or biofilm accumulation	[14,15,16,43,44,45,46,47,48]
Quaternary ammonium polyethylenimine nanoparticles (QPEI NPs)	Immobilized cationic polymer nanoparticles	Densely distributed quaternary ammonium groups interact with and disrupt bacterial membranes	Reduced bacterial viability, adherence, and biofilm formation; antibacterial phase remains largely immobilized	Concentration-dependent increases in solubility and reductions in flexural strength or impact resistance; cytocompatibility and performance depend on loading and dispersion	[49,50,51]
Chitosan and methacrylated chitosan	Cationic biopolymer or polymerizable biopolymer derivative	Interaction of positively charged groups with negatively charged microbial surfaces, leading to altered membrane permeability and compromised membrane integrity; may also function as a carrier	Naturally derived material; chemical modification can improve incorporation into resin networks; experimental formulations show antibacterial and antibiofilm potential	Native chitosan has limited compatibility with hydrophobic methacrylate matrices; mechanical effects vary with concentration; available studies are heterogeneous and predominantly laboratory-based	[52,53,54]

Abbreviations: AgNPs, silver nanoparticles; BAG, bioactive glass; CHX, chlorhexidine; DMAHDM, dimethylaminohexadecyl methacrylate; MDPB, 12-methacryloyloxydodecylpyridinium bromide; QAMs, quaternary ammonium methacrylates; QPEI NPs, quaternary ammonium polyethylenimine nanoparticles; ROS, reactive oxygen species; TiO_2_ NPs, titanium dioxide nanoparticles; ZnO NPs, zinc oxide nanoparticles.

**Table 3:** Representative multifunctional antibacterial systems developed for dental resin composites.

Multifunctional System	Functional Components	Intended Functions	Main Reported Findings	Key Limitations and Design Considerations	References
DMAHDM/NACP composites	Contact-active DMAHDM combined with nanoparticles of amorphous calcium phosphate	Antibacterial activity, reduction in biofilm acid production, calcium and phosphate release, and remineralization	Selected formulations reduced *S. mutans* biofilm activity, acid production, and enamel demineralization adjacent to restoration margins while maintaining the measured flexural properties. Increasing NACP loading enhanced ion release, but performance depended on the proportions of both components.	DMAHDM and NACP concentrations must be balanced because changes in monomer content and filler loading may affect viscosity, filler packing, polymerization, flow, and mechanical properties. Evidence remains predominantly laboratory-based.	[43,44,58]
BAG-containing bioactive composites	Reactive bioactive glass filler incorporated into a methacrylate resin matrix	Antibiofilm activity, ion release, local mineral deposition, and bioactivity	BAG-containing composites promoted calcium phosphate deposition and reduced the viability and thickness of *S. mutans* biofilms.	BAG concentration may influence material properties and cytocompatibility. Freshly cured materials may produce less favorable cellular responses, and preconditioning may alter biological performance.	[36]
MPC/DMAHDM composites	Protein-repellent MPC combined with contact-active DMAHDM	Reduction in protein adsorption and bacterial attachment together with contact-active antibacterial activity	A formulation containing 3 wt.% MPC and 1.5 wt.% DMAHDM reduced protein adsorption, biofilm colony-forming units, metabolic activity, and lactic acid production while maintaining flexural properties under the tested conditions. Protein-repellent and antibacterial activity was retained after water storage for up to 180 days.	MPC is not independently bactericidal, and excessive MPC loading may reduce mechanical properties. Long-term clinical effectiveness and durability under combined biological and mechanical aging remain uncertain.	[48,59,61]
MPC/DMAHDM-containing primer and adhesive systems	Protein-repellent MPC and contact-active DMAHDM incorporated into dental bonding components	Protein repellence, antibacterial activity, and preservation of dentin bonding	Experimental primers and adhesives retained protein-repellent and antibacterial effects and maintained dentin bond strength after six months of water aging.	Findings from bonding systems may not be directly transferable to restorative composites. Performance requires confirmation under salivary pellicle formation, mechanical loading, and clinically relevant multispecies biofilms.	[62]
Rechargeable NACP composites	Rechargeable calcium phosphate filler incorporated into resin matrices with affinity for Ca^2+^ and PO_4_^3−^	Repeated calcium and phosphate recharge and re-release, mineral protection, and prolonged ion delivery	Rechargeable composites underwent repeated recharge and re-release cycles without progressive loss of ion output. Resin-matrix composition and NACP loading strongly influenced recharge capacity, ion release, flow, and mechanical properties.	Recharge requires exposure to an external calcium- and phosphate-containing solution. Clinical recharge protocols, patient compliance, long-term matrix stability, and the relationship between ion release and lesion prevention remain unresolved.	[58,63,64]
Rechargeable DMAHDM/NACP composites	Contact-active DMAHDM combined with rechargeable NACP	Antibacterial activity, reduction in biofilm acid production, and renewable calcium and phosphate delivery	A composite containing 3 wt.% DMAHDM and 20 wt.% NACP maintained ion release through 12 recharge and re-release cycles over six months and reduced biofilm colony-forming units by approximately four orders of magnitude compared with the control.	The reported findings are based on laboratory models. The durability of rechargeability, antibacterial activity, mechanical properties, and biological safety under clinical service requires validation.	[25]
pH-responsive NACP sealants with or without DMAHDM	Acid-responsive NACP, alone or combined with DMAHDM	Increased Ca^2+^ and PO_4_^3−^ release under acidic conditions, mineral protection, and optional contact-active antibacterial activity	NACP-containing sealants reduced mineral loss during pH cycling. A 20 wt.% NACP formulation provided a favorable balance among ion release, rechargeability, flexural properties, and flow, whereas 30 wt.% NACP significantly reduced flowability.	The response is based on passive filler dissolution rather than externally controlled release. Antidemineralization does not necessarily indicate direct biofilm inhibition, and high filler loading may impair handling properties.	[58,65]

Abbreviations: BAG, bioactive glass; DMAHDM, dimethylaminohexadecyl methacrylate; MPC, 2-methacryloyloxyethyl phosphorylcholine; NACP, nanoparticles of amorphous calcium phosphate.

## Data Availability

No new data were created or analyzed in this study. Data sharing is not applicable.
